# Protective role of *SIRT1* (rs3758391 T > C) polymorphism against T2DM and its complications: Influence on GPx activity

**DOI:** 10.1002/hsr2.70106

**Published:** 2024-10-28

**Authors:** Rozita Naseri, Farnaz Khalili, Zohreh Rahimi, Kheirolah Yari, Mansour Rezaei

**Affiliations:** ^1^ Department of Internal Medicine Medical School Kermanshah University of Medical Sciences Kermanshah Iran; ^2^ Department of Clinical Biochemistry Medical School Kermanshah University of Medical Sciences Kermanshah Iran; ^3^ Medical Biology Research Center, Health Technology Institute Kermanshah University of Medical Sciences Kermanshah Iran; ^4^ Department of Biostatistics Medical School Kermanshah University of Medical Sciences Kermanshah Iran

**Keywords:** antioxidants, diabetic neuropathy, diabetic retinopathy, SIRT1 gene variants, T2DM

## Abstract

**Background and Aims:**

Sirtuin‐1 (SIRT1) has antidiabetic effects through the regulation of insulin secretion and modulation of inflammation. The *SIRT1* rs3758391 gene polymorphism affects the level of SIRT1. The current study aimed to investigate the possible influence of *SIRT1* gene variants in relation to oxidative stress parameters on the susceptibility to type 2 diabetes mellitus (T2DM) and its microvascular complications.

**Methods:**

In this case‐control study 398 individuals including 300 patients with T2DM (100 T2DM without complication, 100 diabetic neuropathy patients and 100 patients with diabetic retinopathy) and 98 healthy subjects were studied for *SIRT1* rs3758391 T > C variants. Also, the glutathione peroxidase (GPx) activity and the levels of glutathione (GSH), malondialdehyde (MDA), total antioxidant capacity (TAC), and total oxidative status (TOS) were determined by colorimetric methods. *SIRT1* genotypes were detected using the polymerase chain reaction‐restriction fragment length polymorphism method.

**Results:**

The C allele of *SIRT1* reduced the risk of T2DM, diabetic neuropathy and diabetic retinopathy. Significantly lower levels of GSH, GPx, and TAC were found in diabetic patients compared to control group. However, the level of MDA was significantly higher in patients compared to healthy individuals. Considering all individuals, the GPx activity increased in the presence of the *SIRT1* CC, and TC genotypes compared to the TT genotype. Among all studied individuals the activity of GPx was significantly higher in normal body mass index (BMI) subjects than overweight, and obese individuals. However, among overweight and obese diabetic, diabetic retinopathy and diabetic neuropathy patients the mean level of TOS was significantly higher compared to patients with normal BMI.

**Conclusions:**

Our findings suggest a protective role for *SIRT1* C allele against T2DM and diabetic neuropathy and diabetic retinopathy. We found in the presence of this allele the GPx activity increased. Also, we detected an enhanced oxidative stress level among overweight and obese patients with diabetes and its complications that could be involved in the pathogenesis of the disease.

## 1 INTRODUCTION

Diabetes mellitus (DM), a metabolic disease, results from chronic increased blood glucose (hyperglycemia) due to defect in insulin secretion, the function of insulin or both. In DM patients, the rate of morbidity and mortality increase due to microvascular (retinopathy, neuropathy, and nephropathy) and macrovascular complications (peripheral artery disease, cardiovascular disease, and stroke).^1^, ^2^ Diabetic neuropathy affects 30−50% of DM patients. The metabolic and microvascular consequences of diabetic neuropathy are slowly developed. Both peripheral and central nervous system are affected by the disease. The symptoms of the disease include the paresthesia, and losing the sense of pain, temperature, and touch.^3^ The onset of diabetic neuropathy has not fully been understood, so research on the mechanisms involved in the onset of diabetic neuropathy with focusing on the biomolecules affecting inflammation and oxidative stress is necessary.

Diabetic retinopathy is the most common microvascular complication of diabetes^4^ and is the major cause of acquired adults blindness. Chronic hyperglycemia results in oxidative stress elevation that activates the pathways of protein kinases and polyol with consequences of neuronal and vascular damage. The silent information regulator 2, sirtuins (*SIRT1*) is expressed in the retina, and increased its expression has a protective effect against various ocular diseases such as retina degeneration, cataracts, and optic neuritis.^5^ Sirtuins consist of a family of histone deacetylases that deacetylate histone and none histone lysine residues.^4^ These proteins are encoded by sirtuin genes (*SIRT1* through *SIRT7*), *SIRT1* is the first one that was discovered.^6^ *SIRT1* is known as a longevity gene that encodes a nicotinamide adenine dinucleotide‐dependent class III histone deacetylase.^7^ The SIRT1 is involved in regulating various cellular functions including glucose and lipid metabolism, insulin secretion from the pancreas, mitochondrial biogenesis, inflammation, stress resistance, apoptosis and silencing of chromatin.^8^ SIRT1 can exert antidiabetic effects through the regulation of insulin secretion, insulin resistance improvement, and the modulation of inflammation, and it could be considered as a novel target for treatment of type 2 DM (T2DM).^8^

The *SIRT1* gene consists of 11 exons, and spans 40.7 kb, and is located on chromosome 10q21.3. The *SIRT1* gene contains frequent single nucleotide polymorphisms, some of which are linked with blood pressure.^9^ The *SIRT1* rs3758391 T > C is located in the promoter of *SIRT1* gene and the frequency of its minor allele is 0.146‐0.494.^10^ The CC genotype of *SIRT1* rs3758391 T > C was associated with lower SIRT1 production.^11^

The *SIRT1* rs3758391has been associated with T2DM in various populations.^12^ However, the role of *SIRT1* rs3758391 variants in susceptibility to T2DM complications, including diabetic neuropathy and diabetic retinopathy have not been studied. This polymorphism and other gene variants of the *SIRT1* have been studied in other complications of diabetes. Association between *SIRT1* gene polymorphisms and diabetic nephropathy has been reported and suggested implication of sirtuin‐1 (SIRT1) in the initiation of diabetic nephropathy.^13^ Among Chinese Han population with T2DM, two polymorphisms of the *SIRT1* (rs16924934 and rs3818291) were associated with susceptibility to coronary heart disease (CHD).^14^ Also, it has been suggested that the polymorphism of *SIRT1* rs7896005 might be involved in the risk of CHD in T2DM.^6^ Results of a meta‐analysis indicated the *SIRT1* rs12778366 is negatively associated with diabetic foot ulcer.^15^ Peng et al. study did not find an association between the *SIRT1* rs3758391 polymorphism with T2DM and diabetic foot susceptibility.^9^

The human body naturally defends against the adverse effects of reactive oxygen species (ROS) by both enzymatic and nonenzymatic antioxidant systems.^16^

The glutathione peroxidase (GPx), an antioxidant enzyme, has important role in the metabolism of oxidative stress products.^16^ Obesity, a major driver of T2DM, is a chronic low grade inflammation with permanently enhanced oxidative stress that, due to depletion of enzymatic (GPx, superoxide dismutase and catalase) and nonenzymatic antioxidants (vitamins), obese individuals are more susceptible to oxidative damage.^17^ SIRT1 has an important role in the regulation of oxidant/antioxidant signaling pathways, and a wide variety of genes related to antioxidant.^18^ *SIRT1* gene polymorphism might alter its expression or function contributing to different disorders including neural or vascular lesions.^17^ Increased *SIRT1* gene expression has been associated with total antioxidant status in T2DM patients.^19^

Regarding the complex pathogenesis of diabetes complications and the role of inflammation, and oxidative stress parameters in the pathogenesis of these complications, more research on the gene variants of *SIRT1,* a gene involved in the regulation of metabolism, inflammation and oxidative stress could shed light to the mechanism of diabetes complications. Regarding the absence of a study about the distribution of *SIRT1* gene variants and its association with T2DM complications, the current study aimed to investigate the possible influence of *SIRT1* gene variants in relation to oxidative stress parameters in susceptibility to T2DM and its complications. Also, we investigated the influence of obesity on oxidative stress parameters (GPx activity, total oxidative status [TOS]) in studied individuals.

## 2 METHODS

### 2.1 Study population

In this case‐control study, 300 T2DM patients with a mean age of 56.8 ± 5.6 years, including 100 patients without complication (35 males and 65 females), 100 T2DM with neuropathy (17 males and 83 females) and 100 diabetic retinopathy patients (28 men and 72 women) and 98 healthy individuals (48 men and 50 women) with the mean age of 52.8 ± 7.3 years from the Diabetes Research Center of Taleghani Hospital in Kermanshah were studied. The duration of diabetes in studied individuals was at least 5 years.^1^ The present study was approved by the Ethics Committee of Kermanshah University of Medical Sciences, and written informed consent was provided from each individual (IR.KUMS.REC.1397.529).

### 2.2 Biochemical analysis

Using the method of ferric reducing ability of plasma, the plasma level of total antioxidant capacity (TAC) was measured at 593 nm as previously described.^20^

A colorimetric method was used for detection of TOS level through ferric‐xylenol orange assay. Lipid peroxidation was evaluated by measuring malondialdehyde (MDA) and plasma reduced glutathione (GSH) was detected using the fluorescent reagent of O‐ phthalaldehyde. The ratio of TOS to TAC was used for definition of oxidative stress index. The activity of GPx (U/mgHb) was measured using the Randox kit.^21^

Triglycerides (TG), total plasma cholesterol (TC), and high density lipoprotein‐cholesterol (HDL‐C) levels were determined by the standard enzymatic method (Pars Azmoon kit). Total cholesterol‐(HDL‐C + TG/5) formula was used for calculation of plasma level of low density lipoprotein‐cholesterol (LDL‐C).^12^

### 2.3 Extraction of DNA and genotyping

Using phenol–chloroform method genomic DNA was extracted from ethylenediaminetetraacetic acid‐treated whole blood.^20^

The polymorphism of *SIRT1* rs3758391 T > C was identified by the method of polymerase chain reaction‐restriction fragment length polymorphism (PCR‐RFLP). The designed primers of 5´‐ TGG CCA GAA CCC ATA CTA GG‐3´ as forward primer and the reverse primer of 5´‐ AGC CCT TCC ACT TTC CTC TC‐3' were used for amplification of the gene polymorphism.^22^ The restriction enzyme of Styl was used for digestion the 205‐bp PCR product. The *SIRT1* CC genotype was identified using the presence of 205‐bp band. The *SIRT1* TT genotype was detected with the presence of 120‐ and 85‐bp fragments. In the presence of TC genotype, 3 fragments of 205‐, 120‐, and 85‐bp were observed.

### 2.4 Statistical analysis

The significance of difference in the frequencies *SIRT1* rs3758391 T > C genotypes and alleles between studied groups were calculated using the *χ* ^2^ test. To estimate an association between the *SIRT1* gene variants with the risk of diabetes, and microvascular complications of diabetes, the Odds ratios (OR) and 95% confidence intervals (95% CIs) were calculated using SPSS logistic regression software. Two‐tailed Student's *t*‐test and the analysis of variance were used for comparing quantitative data between groups. *p* < 0.05 was considered as statistically significant. Box plots were used to visualize the distribution comparison of some parameters between groups.

## 3 RESULTS

Table 1 indicates the characteristics of diabetic and healthy individuals. The body mass index (BMI) was significantly higher in patients (29.0 ± 4.3 kg/m^2^) compared to controls (26.9 ± 4.2 kg/m^2^, *p* < 0.001). Significantly lower levels of GSH (9.9 ± 4.1 nmol/mL, *p* = 0.001), GPx (46.4 ± 10.5 U/mgHb, *p* < 0.001) and TAC (742.8 ± 131.2 nmol/mL, *p* = 0.046) were detected in all diabetic patients compared to controls (11.3 ± 3.4 nmolml, 59.9 ± 10.9 U/mgHb, and 770.2 ± 113.7 nmol/mL, respectively). The TOS level was significantly higher in controls than patients (4.2 ± 1.2 vs. 3.8 ± 2, *p* = 0.023). However, in T2DM patients the level of MDA was significantly higher (6.8 ± 2.1 nmol/mL, *p* < 0.001) than that in healthy individuals (5.1 ± 1.6 nmol/mL) (Table 1). The mean levels of fasting blood sugar and HbA1_C_ in all diabetic patients were 170.3 ± 71.2 mg/dL, and 8.82 ± 3.9%, respectively. Plasma levels of lipids in various groups of diabetic patients have been compared and presented in Table 2.

**Table 1:** Characteristics of patients and controls.

Variables	Patients (*n* = 300) Mean ± SD	Controls (*n* = 98) Mean ± SD	*p* Value
Age (years)	56.8 ± 5.6	52.8 ± 7.3	<0.001
BMI (Kg/m^2^)	29.0 ± 4.3	26.9 ± 4.2	<0.001
Glutathione (nmol/mL)	9.9 ± 4.1	11.3 ± 3.4	0.001
GPx (U/mgHb)	46.4 ± 10.5	59.9 ± 10.9	<0.001
TAC (nmol/mL)	742.8 ± 131.2	770.2 ± 113.7	0.046
TOS (nmol/mL)	3.8 ± 2.0	4.2 ± 1.2	0.023
MDA (nmol/mL)	6.8 ± 2.1	5.1 ± 1.6	<0.001
OSI (TOS/TAC)	0.0052 ± 0.0028	0.0055 ± 0.0017	0.240

Abbreviations: BMI, body mass index; GPx, glutathione peroxidase; MDA, malondialdehyde; OSI, oxidative stress index; TAC, total antioxidant capacity; TOS, total oxidative status.

**Table 2:** Comparing plasma lipid levels in T2DM patients without complication, diabetic neuropathy, and diabetic retinopathy.

Variables	T2DM without complication (*n* = 100) Mean ± SD	T2DM with neuropathy (*n* = 100) Mean ± SD	T2DM with retinopathy (*n* = 100) Mean ± SD	*p* Value
Cholesterol (mg/dL)	155.7 ± 31.3	163.8 ± 32.5	156.9 ± 32.1	0.09
Triglycerides (mg/dL)	136.1 ± 75.7	155.1 ± 63.3	143.3 ± 62.3	0.13
HDL‐C (mg/dL)	41.0 ± 4.5	40.1 ± 8.7	39.5 ± 10.4	0.52
LDL‐C (mg/dL)	86.4 ± 26.3	88.8 ± 22.4	82.5 ± 23.6	0.17

Abbreviations: HDL‐C, high density lipoprotein‐cholesterol; LDL, low density lipoprotein‐cholesterol.

Distribution of the *SIRT*1 genotypes (rs3758391 T > C) was in Hardy‐Weinberg equilibrium in all T2DM patients (*χ* ^2^ = 2.33, *p* > 0.1), in T2DM patients without complication (*χ* ^2^ = 2.04, *p* > 0.1), in T2DM patients with neuropathy (*χ* ^2^ = 0.92, *p* > 0.1) and in T2DM patients with retinopathy (*χ* ^2^ = 0.08, *p* > 0.1). Table 3 demonstrates the *SIRT1* genotypes and alleles frequencies among all diabetic patients compared to control group. A significantly lower frequency of the *SIRT1* CC genotype in all diabetic patients (27.3%, *p* < 0.001) than in controls (36.7%) indicated its protective role against diabetes. Also, a lower frequency of the C allele of *SIRT1* was observed in patients (54.3%, *p* = 0.001) compared to controls (68.4%) that decreased the risk of T2DM by 45% (OR = 0.55, *p* = 0.001) (Table 3).

**Table 3:** Distribution of SIRT1 (rs3758391 T > C) genotypes and alleles in patients and controls.

SIRT1	All patients (*n* = 300) *n* (%)	*χ* ^2^, *p* Value	OR, *p* Value	Controls (*n* = 98) *n* (%)
*Genotypes*				
TT	56 (18.7)			0 (0)
TC	162 (54)	19.9, <0.001^a^		62 (63.3)
CC	82 (27.3)	21.5, <0.001^a^		36 (36.7)
*Alleles*				
T	274 (45.7)			62 (31.6)
C	326 (54.3)	11.93, 0.001	0.55 (95% CI 0.39−0.77), 0.001	134 (68.4)

*Note*: *χ* ^2^ = 21.6, *p* < 0.001 comparing 3 genotypes between patients and controls.

Abbreviation: SIRT1, Sirtuin‐1.

a Compared to TT genotype.

In Table 4 distribution of genotypes and alleles of *SIRT1* in three groups of T2DM patients including T2DM without complication, diabetic neuropathy, and diabetic retinopathy patients are demonstrated. The frequencies of the C allele in diabetic patients without complication, diabetic neuropathy and diabetic retinopathy were 52.5, 56 and 54.5%, respectively. The presence of the C allele decreased the risk of T2DM without complication risk by 49%, reduced the risk of diabetic neuropathy by 41% (OR = 0.59, *p* = 0.01), and decreased the diabetic retinopathy risk by 45% (OR = 0.55, *p* = 0.005).

**Table 4:** Distribution of SIRT1 (rs3758391 T > C) genotypes and alleles in T2DM patients without complication, diabetic neuropathy, diabetic retinopathy and controls.

SIRT1		T2DM without complication (*n* = 100), *n* (%)	T2DM with neuropathy (*n* = 100), *n* (%)	T2DM with retinopathy (*n* = 100), *n* (%)	Controls (*n* = 98), *n* (%)
Genotypes	TT	19 (19)	17 (17)	20 (20)	0 (0)
	TC	57 (57)	54 (54)	51 (51)	62 (63.3)
	CC	24 (24)	29 (29)	29 (29)	36 (36.7)
		*χ* ^2^ = 20.9, *p* < 0.001^a^	*χ* ^2^ = 16.8, *p* < 0.001^a^	*χ* ^2^ = 19.2, *p* < 0.001^a^	
Alleles	T	95 (47.5)	88 (44)	91 (45.5)	62 (31.6)
	C	105 (52.5)	112 (56)	109 (54.5)	134 (68.4)
		*χ* ^2^ = 10.4, *p* = 0.001 OR = 0.51, (95%CI [0.34−0.77])	*χ* ^2^ = 6.43, *p* = 0.01 OR = 0.59, (95%CI [0.39−0.88])	*χ* ^2^ = 8.03, *p* = 0.005 OR = 0.55, (95%CI [0.37−0.84])	

*Note*: *χ* ^2^ = 22.8, *p* = 0.001 comparing three genotypes between four groups.

Abbreviation: SIRT1, Sirtuin‐1.

a Comparing CC with TT genotype between patients and controls.

Considering all individuals (patients and controls) the activity of GPx was significantly higher in the presence of *SIRT1* CC (50 ± 11.9 U/mgHb, *p* = 0.027), and TC genotypes (50.7 ± 12.4, *p* = 0.004) than TT genotype (45 ± 10.6 U/mgHb).

**Figure 1:**
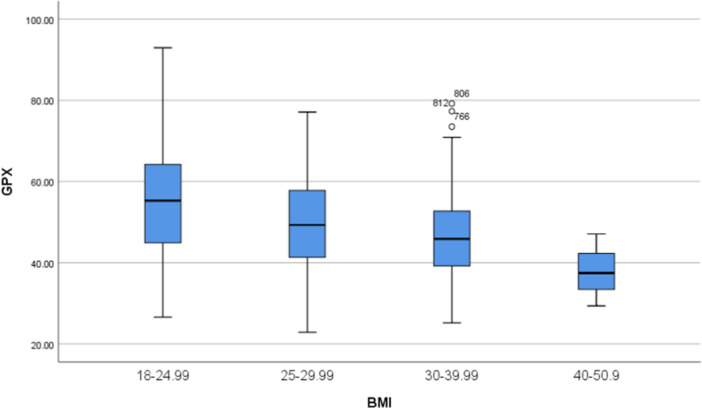
Box Plot distribution of GPx according to body mass index (BMI) among all studied individuals. GPx, glutathione peroxidase.

Considering all individuals, the activity of GPx was significantly higher (55.1 ± 13.1 U/mgHb) in normal BMI individuals (18.5‐24.9 Kg/m^2^) compared with obese subjects, BMI ≥ 30 Kg/m^2^, (46.6 ± 10.9 U/mgHb, *p* < 0.001), with overweight subjects, BMI 25−29.9 Kg/m^2^ (49.9 ± 11.8 U/mgHb, *p* = 0.003), and with overweight and obese individuals, BMI ≥ 25 Kg/m^2^ (48.5 ± 11.5, *p* < 0.001) (Figure 1).

**Figure 2:**
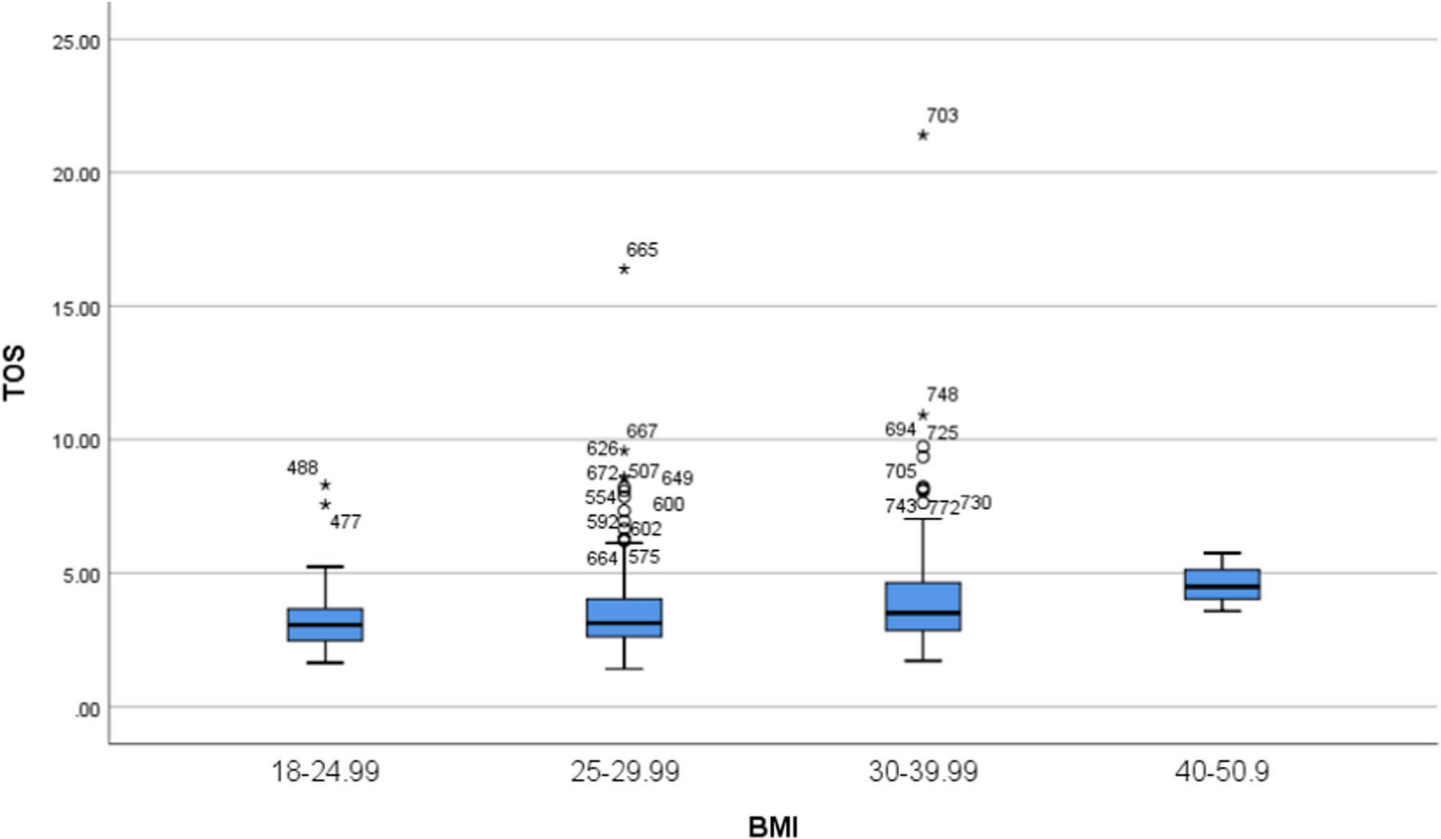
Box Plot distribution of total oxidative status (TOS) according to BMI among all patients. BMI, body mass index.

The TOS level of all overweight and obese diabetic patients was significantly higher than that of normal BMI patients (3.9 ± 2.1 vs. 3.3 ± 1.3 nmol/mL, *p* = 0.013). Figure 2 indicates Box Plot distribution of TOS according to BMI among all patients. Also, overweight and obese diabetic retinopathy and diabetic neuropathy patients had significantly higher TOS levels than normal BMI patients (3.9 ± 2.7 vs. 3.1 ± 0.76 nmol/mL, *p* = 0.022 and 4.3 ± 2.2 vs. 3.5 ± 0.96 nmol/mL, *p* = 0.037, respectively).

## 4 DISCUSSION

T2DM is the most prevalent metabolic disease that is associated with hyperglycemia, hypertension, and dyslipidemia.^23^ The ROS synthesis, glycation, various inflammatory cytokines, the polyol pathway and alterations in the activity of protein kinase C are some mechanisms play role in the development of diabetic neuropathy.^3^

Diabetic retinopathy is a multifactorial disease that occurs primarily through the long‐term adverse effects of hyperglycemia. Neurons, glial cells, and vascular elements of the retina are affected by diabetic retinopathy.^4^

SIRT1 protein has antidiabetic effects through the regulation of insulin secretion and modulation of inflammation.^8^ So, SIRT1 could be considered as a novel therapeutic target for T2DM.^21^ SIRT1 has a role as the energy sensor of cells that links metabolic stress with cellular response. The *SIRT1* gene is downregulated in the retina of diabetic patients.^5^

The *SIRT1* rs3758391 polymorphism locates in the promoter region on chromosome 10q21.3, within a putative binding site of p53. The presence of the C allele of this polymorphism compared to the T allele results in a lower binding affinity for p53, suggesting a functional role for this variant. The CC genotype of this polymorphism was associated with lower SIRT1 production. It has been indicated that the TT and TC genotypes of this polymorphism are associated with elevated *SIRT1* expression in the skeletal muscle of overweight subjects.^11^ In a study among T2DM patients, the gene expression of *SIRT1* increased by 1.8‐fold in the presence of the rs3758391 TT genotype in comparison to the CC genotype. So, it was suggested that this polymorphism is associated with the modification of *SIRT1* gene expression.^12^

We observed a significantly lower frequency of *SIRT1* CC genotype and *SIRT1* C allele in diabetic patients than in controls that decreased the risk of T2DM. Also, for the first time, we studied the *SIRT1* variants in T2DM patients with neuropathy and retinopathy. We detected a protective role for the C allele of this polymorphism against diabetic neuropathy and retinopathy. In our study, the frequency of the protective C allele in T2DM (54.3%) was lower than that in the control group (68.4%). In accordance with our study, in a study from Iran, the frequency of the C allele of *SIRT1* was reported to be 79.55% in controls and 54.2% among T2DM patients.^24^ The results of this study confirmed that the T allele of the *SIRT1* rs3758391 is a strong risk factor for T2DM and diabetic nephropathy among a population from southwest Iran.^24^ However, another study from Iran indicated the *SIRT1* rs3758391 polymorphism had a protective role against T2DM in various genetic models of the CT versus CC genotype and the combined genotype of CT + TT versus CC genotype.^23^ Peng et al., indicated that patients with T2DM and diabetic foot exhibited downregulation of *SIRT1* expression; however, they failed to find a significant association between *SIRT1* rs3758391 with T2DM, and with diabetic foot susceptibility.^17^ In a study from Mexico City, a significant association between the T allele of the *SIRT1* rs3758391 with T2DM susceptibility was observed.^25^ Furthermore, Tavakoli et al. reported that diabetic neuropathy was associated with the *SIRT1* rs3758391 TC and TT genotypes.^24^

SIRT1 is involved in the regulation of gluconeogenesis, fatty acid oxidation, lipogenesis, and mitochondrial biogenesis in various tissues,^26^ so SIRT1 deficiency impairs metabolism.^27^ SIRT1 affects lipid metabolism through the activation of some nuclear receptors, such as peroxisome proliferator activated receptor‐α, the receptors of liver X, and farnesoid X and by the sterol regulatory element binding protein down regulation.^8^

In the present study, among patients the activity of GPx and the TAC and GSH levels were significantly less and the MDA level was significantly more than those in the control group. Damage to the antioxidant enzymes, including GPx through protein glycation in the presence of hyperglycemia might results in lower level of GPx.^28^ Based on the results of experimental and clinical studies, T2DM is closely correlated to oxidative stress.^29^ Oxidative stress is involved in insulin resistance, and the TAC level was negatively correlated with the glycemic level in T2DM patients.^30^ Also, increased MDA level, an index of lipid peroxidation, impaired biological structures such as membranes.^30^ Further, enhanced oxidative stress is related to the reduced level and the activity of SIRT1.^30^

We detected that the *SIRT1* variants affected the activity of the GPx with higher GPx activity in the presence of *SIRT1* CC and TC genotypes than TT genotype. There are no studies on the influence of *SIRT1* rs3758391 on the GPx activity in T2DM patients. Sirtuin1 is an epigenetic enzyme that its reduced level is implicated in the development of micro‐ and macro‐vascular complications. Sirtuin1 level is increased by natural antioxidant and anti‐inflammatory compounds and also after treatment with standard anti‐hyperglycemic, anti‐hypertensive, lipid‐lowering and anticoagulant drugs. So, upregulation of SIRT1 could be a potent therapeutic approach for the prevention of the development and progression of diabetic complications.^31^

The oxidative stress due to hyperglycemia results in tissue damage that is recognized as an inflammation by organism. Both obesity and DM are proinflammatory conditions of the body. The presence of subclinical systemic inflammation in obese diabetic patients, increased levels of C–reactive protein, interleukine‐6 (IL‐6), and tumor necrosis factor‐α, predicts the development of T2DM with reduced insulin sensitivity in peripheral tissues.^32^ In a study, the effects of DM duration on the body reparative ability, immune response and the DM complications development were examined. It was found that the physiological age and the duration of DM decreased the repair ability of the body and the immune response was weakened in DM.^33^ In other study,^34^ oxidative stress and IL‐6 levels were examined in the diabetic polyneuropathy. They found the level of IL‐6 was correlated with neuropathy in younger patients with DM for less than 10 years.

Obesity is a chronic low‐grade inflammation with permanently enhanced oxidative stress. The association between obesity and impaired serum glycemic level and progression towards diabetes is through various cellular mechanisms (alterations in insulin signaling, glucose transport and dysfunction of pancreatic β cells), enhanced oxidative stress and inflammation. The coexistence of obesity significantly increased the free radicals and ROS production that are involved in diabetes and diabetic complications pathogenesis.^32^ In the present study, we found significantly lower GPx activity in overweight and obese individuals compared to normal BMI individuals. Also, we observed significantly higher TOS level in all overweight and obese diabetic patients, diabetic retinopathy and neuropathy patients compared to normal BMI patients. Previously, among T2DM patients from Kurdistan of Iraq, we indicated the highest levels of TOS and MDA in obese diabetic patients compared to normal BMI diabetic patients.^35^ We suggested obesity increases oxidative stress through chronic inflammation, ROS production and lipid peroxidation compounds such as MDA and decreased TAC level that are related to the diabetes progression.^35^

## 5 CONCLUSION

In summary, the present study using the PCR‐RFLP method analyzed the gene variants of *SIRT1* rs3758391 in 300 T2DM patients and 98 healthy individuals. Also, by valid biochemical methods we measured oxidative stress parameters in these individuals. For the first time we evaluated the *SIRT1* gene variants in T2DM complications, diabetic neuropathy and retinopathy, and found the *SIRT1* rs3758391 is associated with decreased risk of T2DM, diabetic neuropathy and diabetic retinopathy. Our findings suggest the level of antioxidants of GPx and TAC and GSH reduced and the MDA level increased in diabetic patients than controls. Also, the presence of *SIRT1* variants affected the activity of the GPx with higher GPx activity in the presence of *SIRT1* CC, and TC genotypes than TT genotype. Further, we indicated increased oxidative stress (higher TOS level and lower GPx activity) in overweight and obese diabetic patients, diabetic retinopathy and neuropathy patients compared to normal BMI patients that could be involved in the pathogenesis of diabetes and its complications. The limitation of our study is the lack of studying *SIRT1* gene expression and the plasma level of SIRT1 to detect their possible association with *SIRT1* rs3758391 variants. SIRT1 is an epigenetic enzyme that its level increases by various natural antioxidant and anti‐inflammatory compounds and some drugs. So, future work can be focused on studying *SIRT1* expression in relation to *SIRT1* variants and also investigation the methylation status of *SIRT1* in relation to lifestyle in healthy individuals and in patients with chronic diseases such as diabetes.

## AUTHOR CONTRIBUTIONS

**Rozita Naseri**: Methodology; writing—original draft; investigation. **Farnaz Khalili**: Conceptualization; writing—review and editing; methodology. **Zohreh Rahimi**: Conceptualization; investigation; funding acquisition; writing—original draft; methodology; validation; writing—review and editing; supervision; formal analysis; project administration. **Kheirolah Yari**: Writing—review and editing; conceptualization; methodology; investigation; writing—original draft. **Mansour Rezaei**: Writing—review and editing; software; formal analysis; data curation.

## CONFLICT OF INTEREST STATEMENT

Zohreh Rahimi is an Editorial Board member of Health Science Reports and a coauthor of this article. To minimize bias, they were excluded from all editorial decision‐making related to the acceptance of this article for publication. The remaining authors declare no conflict of interest.

## ETHICS STATEMENT

The study was approved by the Ethics Committee of the Kermanshah University of Medical Science (IR.KUMS.REC.1397.529), and written informed consent was obtained from each individual. All methods were carried out in accordance with relevant guidelines and regulations expressed in the declaration of Helsinki.

## TRANSPARENCY STATEMENT

The lead author Zohreh Rahimi, Kheirolah Yari affirms that this manuscript is an honest, accurate, and transparent account of the study being reported; that no important aspects of the study have been omitted; and that any discrepancies from the study as planned (and, if relevant, registered) have been explained.

## Data Availability

The authors confirm that the data supporting the findings of this study are available within the article [and/or] its supplementary materials.
